# Masked by Aneurysmal Hemorrhage: Herpes Simplex Virus (HSV)-2 Encephalitis in a Patient With Subarachnoid and Intraparenchymal Hemorrhage

**DOI:** 10.7759/cureus.112475

**Published:** 2026-07-11

**Authors:** Tina Tripathi, Lauren Touleyrou

**Affiliations:** 1 Internal Medicine, Wayne State University School of Medicine, Detroit, USA; 2 Infectious Disease, Detroit Medical Center/Wayne State University, Detroit, USA

**Keywords:** encephalitis, herpes simplex virus-2, hsv-2, hsv-2 encephalitis, intracranial hemorrhage and hsv-2

## Abstract

Herpes simplex virus (HSV) encephalitis is a neurologic emergency associated with significant morbidity and mortality. While HSV type 1 is the most common cause in adults, HSV type 2 (HSV-2) is a rare etiology of encephalitis and may present atypically. Intracranial hemorrhage is an uncommon complication of HSV encephalitis and can obscure diagnosis, particularly in critically ill patients with competing neurologic pathologies. Here, we present the case of a 73-year-old female with unknown past medical history who was found unresponsive and admitted with acute subarachnoid and intraparenchymal hemorrhage secondary to a ruptured right internal carotid artery aneurysm. She underwent external ventricular drain placement and endovascular aneurysm coiling. During her neuro-intensive care unit course, she developed recurrent fevers and progressive encephalopathy despite neurosurgical intervention and broad-spectrum antimicrobial therapy. Cerebrospinal fluid obtained from the ventricular drain demonstrated pleocytosis with significant red blood cell contamination, and bacterial cultures remained negative. Polymerase chain reaction (PCR) testing of the cerebrospinal fluid ultimately returned positive for HSV-2. Antibacterial therapy was discontinued, and intravenous acyclovir was initiated. This case highlights the diagnostic challenges of HSV-2 encephalitis in the setting of intracranial hemorrhage, where overlapping clinical features may delay recognition and treatment. HSV-2 encephalitis has been associated with vascular complications, including cerebral vasculitis and hemorrhage, though such presentations remain rare. HSV encephalitis should remain on the differential diagnosis for patients with persistent fever and worsening encephalopathy, even in the presence of an alternative structural neurologic diagnosis. Early consideration of viral etiologies and timely cerebrospinal fluid PCR testing are critical to avoid delays in antiviral therapy and potentially improve outcomes.

## Introduction

Herpes simplex virus (HSV) encephalitis is the most common cause of sporadic, nonendemic viral encephalitis worldwide and represents a neurologic emergency due to its high morbidity and mortality if left untreated [1]. The disease is classically characterized by acute onset of fever, headache, altered mental status, and focal neurologic deficits, often progressing rapidly to seizures, coma, and death. HSV encephalitis is most commonly caused by HSV type 1 (HSV-1) in adults, with predilection for the temporal and frontal lobes, leading to the characteristic necrotizing inflammation seen on neuroimaging and histopathology. 

Prompt recognition and initiation of antiviral therapy with intravenous acyclovir significantly reduces mortality from approximately 70% to 20-30% and improves neurologic outcomes [1-4]. However, diagnosis may be delayed or obscured in critically ill patients with competing neurologic pathologies, atypical cerebrospinal fluid (CSF) findings, or concurrent intracranial hemorrhage. Polymerase chain reaction (PCR) testing of CSF remains the diagnostic gold standard, though interpretation can be challenging in the setting of blood-contaminated CSF or ventricular drainage. 

HSV type 2 (HSV-2) is a less common cause of encephalitis in adults and is more frequently associated with meningitis, recurrent lymphocytic meningitis (Mollaret’s meningitis), and immunocompromised hosts. When HSV-2 encephalitis does occur, it may follow a more indolent or atypical course and has been associated with vascular complications, including cerebral vasculitis and ischemic or hemorrhagic stroke [5]. Intracranial hemorrhage remains a rare but increasingly recognized complication of HSV encephalitis, with Modi et al. citing a prevalence rate of 2.7% [6].

We present a case of HSV-2 encephalitis in an elderly patient with acute subarachnoid and intraparenchymal hemorrhage, whose diagnosis was delayed due to overlapping neurologic insults and critical illness. This case highlights the importance of maintaining a broad differential diagnosis for altered mental status and fever in patients with intracranial hemorrhage and underscores the need for early consideration of viral encephalitis, even in the presence of an alternative structural explanation.

## Case presentation

A 73-year-old female with unknown past medical history was brought to the emergency department after being found unresponsive by emergency medical services during a wellness check, following two days of missed work. During her hospital stay, no additional risk factors were identified.

On arrival, she was febrile and had a Glasgow Coma Scale (GCS) score of 4, prompting emergent intubation for airway protection. 

Initial laboratory evaluation demonstrated a white blood cell count of 4,000 cells/μL, acute kidney injury (creatinine 1.99 mg/dL), and elevated creatine phosphokinase (CPK) (1,923 U/L) (Table 1). The CPK value indicates that the patient was undergoing muscle injury consistent with early or mild rhabdomyolysis, likely causing some renal impairment. 

**Table 1:** Labs on Admission RBC = red blood cell count; WBC = white blood cell count; CPK = creatine phosphokinase

Lab	Lab Value
WBC	4,000 cells/μL (normal range: 4,500-11,000 cells/μL)
Hemoglobin	12.3 g/dL (normal range for female: 12.0-16.0 g/dL)
Hematocrit	37.4% (normal range for female: 36%-46%)
Platelet Count	189,000/μL (normal range: 150,000-400,000/μL)
Creatinine	1.99 mg/dL (normal range: 0.6-1.2 mg/dL)
CPK	1,923 U/L (normal range: 22-198 U/L)

**Figure 1:**
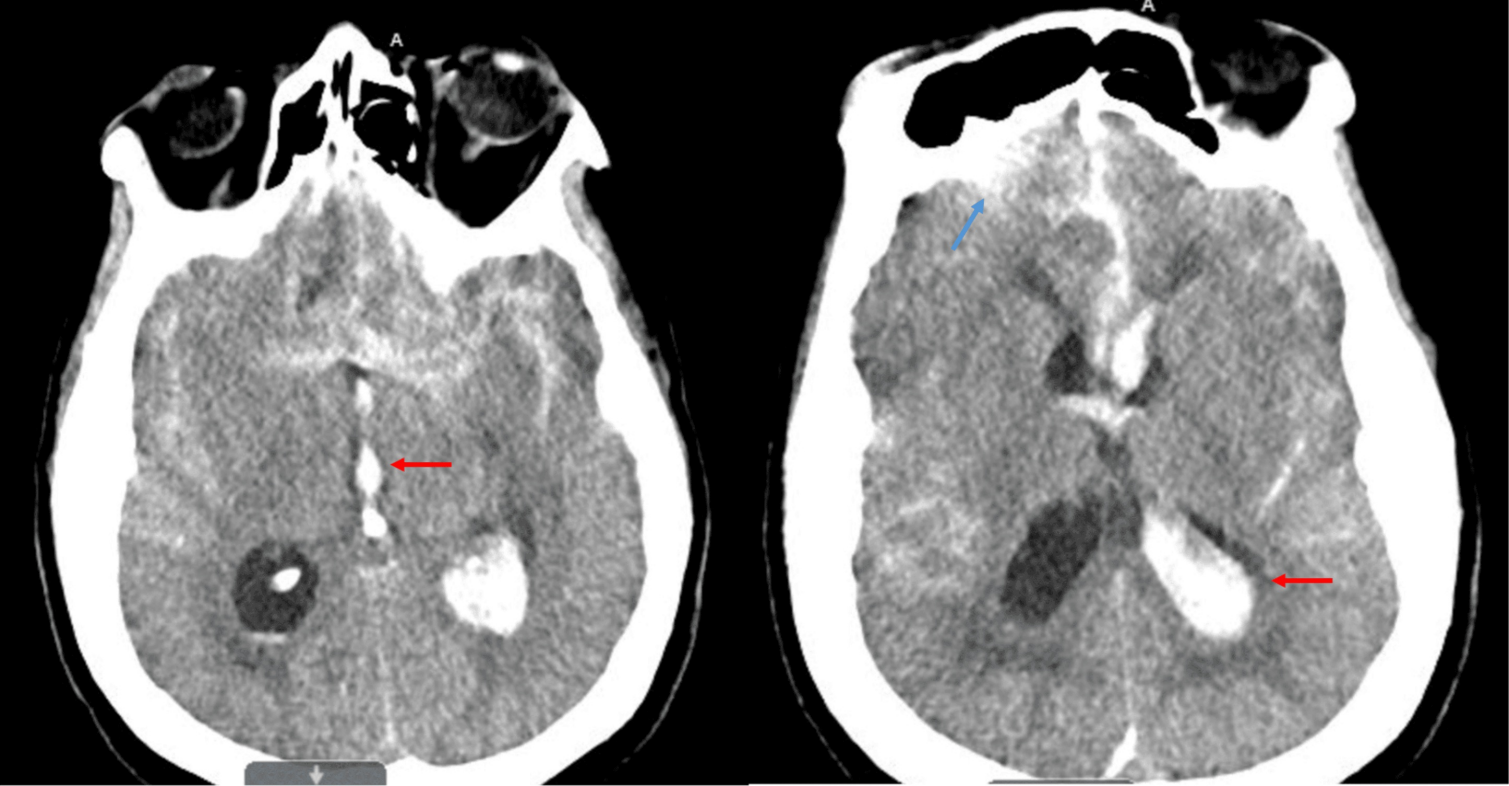
Head CT of patient Head CT of 73-year-old female who was found unresponsive by EMS with a low level of consciousness. There is evidence of extensive subarachnoid hemorrhage in basilar cisterns with extension into the ventricles (red arrows). Also pictured is a 2.4 x 2 x 1.3 cm intraparenchymal hemorrhage in the right frontal lobe (blue arrow).

Non-contrast computed tomography (CT) of the head revealed extensive subarachnoid hemorrhage within the basilar cisterns with intraventricular extension, as well as a 2.4 × 2.0 × 1.3 cm right frontal intraparenchymal hemorrhage (Figure 1). This qualifies as a Grade IV on the modified Fisher Grading Scale [7] for subarachnoid hemorrhage.

An external ventricular drain (EVD) was placed emergently, and subsequent cerebral angiography demonstrated a ruptured right internal carotid artery aneurysm measuring 5.6 × 4.5 mm, which was treated with endovascular coiling. 

During the initial hospital course, the patient remained critically ill in the neuro-intensive care unit (NICU). On hospital day five, she developed recurrent fevers with temperatures up to 38.7°C and worsening mental status on serial neurologic examinations. A septic workup was initiated same-day, and Infectious Disease consultation was obtained. Empiric antimicrobial therapy with vancomycin pharmacy-dosed and intravenous piperacillin-tazobactam 4.5g every six hours was started and later broadened to include cefepime. 

CSF obtained from the EVD demonstrated a nucleated cell count of 374 cells/μL and a red blood cell count of 26,000 cells/μL on hospital day seven (Table 2).

**Table 2:** CSF Studies CSF = cerebrospinal fluid; RBC = red blood cells

Lab	Lab Value
Appearance (fluid)	Cloudy
Color (fluid)	Red (normal: clear)
CSF RBC	26,000 cells/μL (normal range: 0-5 cells/μL)
CSF Nucleated Cells	374 cells/μL (normal range: 0-5 cells/μL)
Neutrophils % (fluid)	74%
Lymphocytes % (fluid)	8%
Monocytes % (fluid)	18%
Eosinophils % (fluid)	None Seen
Basophils % (fluid)	None Seen
Blasts % (fluid)	None Seen
Other Cells (Fluid)	None Seen
Cells Counted	100

CSF bacterial cultures remained negative. Due to concern for cefepime-associated neurotoxicity, antimicrobial therapy was transitioned to ceftriaxone (day 8). Given persistent encephalopathy and fevers despite antibacterial therapy, CSF viral PCR testing was pursued and returned positive for HSV-2. Antibacterial agents were discontinued, and intravenous acyclovir 8mg/kg every 12 hours was initiated for 14 days. 

## Discussion

HSV encephalitis is a neurologic emergency associated with significant morbidity and mortality, even in the era of effective antiviral therapy. Although HSV-1 is responsible for the majority of adult cases, HSV-2 is a less common cause of encephalitis and more frequently presents as meningitis or recurrent lymphocytic meningitis. When HSV-2 encephalitis does occur, it may follow an atypical clinical course and has been associated with vascular complications, including cerebral vasculitis, ischemic stroke, and intracranial hemorrhage [5]. 

The diagnosis of HSV encephalitis is typically supported by characteristic neuroimaging findings involving the temporal lobes and by CSF abnormalities, including lymphocytic pleocytosis, elevated protein, and normal glucose levels. However, these diagnostic features may be absent or difficult to interpret in critically ill patients with concurrent intracranial pathology. In our patient, the presence of aneurysmal subarachnoid hemorrhage and intraparenchymal hemorrhage provided an alternative explanation for altered mental status and fever, complicating the diagnostic evaluation. Additionally, CSF interpretation was limited by significant red blood cell contamination from the EVD, underscoring the challenges of diagnosing viral encephalitis in the neurocritical care setting. A second lumbar puncture was not pursued because the patient's clinical course secondary to HSV was found to be highly probable despite the risks of the CSF findings potentially being a false positive or due to an incidental shedding of viral particles. Other root causes of sepsis with the positive CSF results were considered, however the positive CSF result leaned the physicians towards HSV encephalitis as the diagnosis. 

Intracranial hemorrhage is a rare but increasingly recognized complication of HSV encephalitis. Pathologic studies typically demonstrate necrotizing inflammation with scattered microhemorrhages; however, clinically significant intraparenchymal or subarachnoid hemorrhage is uncommon and most often occurs during the second week of illness. Several mechanisms have been proposed to explain hemorrhagic complications, including direct viral invasion of cerebral vessels, immune-mediated vasculitis, endothelial injury, and rupture of fragile vessels in the setting of elevated intracranial pressure. Prior reports suggest that HSV-2-associated hemorrhagic and ischemic complications may be more frequently related to large-vessel vasculitis, whereas HSV-1 is more often associated with vessel disruption and necrosis, which may portend a worse prognosis [2,5].

## Conclusions

This case highlights the importance of maintaining a broad differential diagnosis for fever and progressive encephalopathy in patients with intracranial hemorrhage. In the neuro-intensive care unit, fever is commonly attributed to central fever, bacterial infection, or postoperative inflammation; however, persistent fevers accompanied by worsening mental status should prompt consideration of viral encephalitis, even when an alternative structural diagnosis is present. Early involvement of infectious disease specialists and timely CSF viral PCR testing are essential, as delays in initiation of acyclovir therapy are associated with worse neurologic outcomes. 

In summary, this case contributes to the limited literature describing HSV-2 encephalitis complicated by intracranial hemorrhage and emphasizes the diagnostic challenges posed by overlapping neurologic pathologies. While in this specific case causality between HSV-2 and the hemorrhagic findings cannot be definitively established due to the confirmed ruptured aneurysm, it supports that the diagnosis of HSV-2 encephalitis was delayed and masked by aneurysmal hemorrhage and critical illness. Heightened clinical awareness of this rare presentation may facilitate earlier diagnosis and treatment, potentially improving outcomes in similar patients.
